# Crystal structure, Hirshfeld surface analysis and DFT studies of 1-benzyl-3-[(1-benzyl-1*H*-1,2,3-triazol-5-yl)meth­yl]-2,3-di­hydro-1*H*-1,3-benzo­diazol-2-one monohydrate

**DOI:** 10.1107/S2056989019016876

**Published:** 2020-01-01

**Authors:** Asmaa Saber, Nada Kheira Sebbar, Tuncer Hökelek, Mohamed Labd Taha, Joel T. Mague, Noureddine Hamou Ahabchane, El Mokhtar Essassi

**Affiliations:** a Laboratoire de Chimie Organique Hétérocyclique URAC 21, Pôle de Compétence Pharmacochimie, Av. Ibn Battouta, BP 1014, Faculté des Sciences, Université Mohammed V, Rabat, Morocco; b Laboratoire de Chimie Appliquée et Environnement, Equipe de Chimie Bioorganique Appliquée, Faculté des Sciences, Université Ibn Zohr, Agadir, Morocco; c Department of Physics, Hacettepe University, 06800 Beytepe, Ankara, Turkey; d Department of Chemistry, Tulane University, New Orleans, LA 70118, USA

**Keywords:** crystal structure, di­hydro­benzo­diazole, hydrogen bond, triazole, π-stacking, Hirshfeld surface

## Abstract

In the title mol­ecule, C_24_H_21_N_5_O·H_2_O, the di­hydro­benzo­diazole moiety is not quite planar, while the whole mol­ecule adopts a U-shaped conformation in which there is a close approach of the two benzyl groups. In the crystal, chains of alternating mol­ecules and lattice water extending along [201] are formed by O—H_UncoordW_⋯O_Dhyr_ and O—H_UncoordW_⋯N_Trz_ (UncoordW = uncoordinated water, Dhyr = di­hydro and Trz = triazole) hydrogen bonds. The chains are connected into layers parallel to (010) by C—H_Trz_⋯O_UncoordW_ hydrogen bonds with the di­hydro­benzo­diazole units in adjacent layers inter­calating to form head-to-tail π-stacking [centroid-to-centroid distance = 3.5694 (11) Å] inter­actions between them, which generates the overall three-dimensional structure. Hirshfeld surface analysis indicates that the most important contributions for the crystal packing are from H⋯H (52.1%), H⋯C/C⋯H (23.8%) and O⋯H/H⋯O (11.2%) inter­actions. Hydrogen-bonding and van der Waals inter­actions are the dominant inter­actions in the crystal packing. Density functional theory (DFT) optimized structures at the B3LYP/ 6–311 G(d,p) level are compared with the experimentally determined mol­ecular structure in the solid state. The HOMO–LUMO behaviour was elucidated to determine the energy gap.

## Abstract 2

The di­hydro­benzo­diazole moiety is not quite planar while the whole mol­ecule adopts a U-shaped conformation in which there is a close approach of the two benzyl groups. Chains of alternating mol­ecules and lattice water extending along the normal to (301) are formed by O—H⋯O and O—H⋯N hydrogen bonds.

## Chemical context   

Nitro­gen heterocyclic compounds are known to exhibit excellent biological and pharmaceutical activities (Olesen *et al.*, 1994 ▸; Baxter & Clarke, 1992 ▸; Saber *et al.*, 2020 ▸; Rémond *et al.*, 1997 ▸). The benzimidazole core has several active sites and provides great responsiveness, making it an excellent heterocyclic precursor in the syntheses of the new heterocyclic compounds (Saber *et al.*, 2018*a* ▸,*b* ▸; Ouzidan *et al.*, 2011 ▸; Saber *et al.*, 2020 ▸). With respect to the biological applications of benzimidazolone derivatives, it has been shown that these compounds are found to possess potent anti­oxidant (Gaba *et al.*, 2014 ▸), anti­parasitic (Ayhan-Kılcıgil *et al.*, 2007 ▸), anthelmintic (Navarrete-Vazquez *et al.*, 2001 ▸), anti­proliferative (Ravina *et al.*, 1993 ▸), anti-HIV (Garuti *et al.*, 2000 ▸), anti­convulsant (Rao *et al.*, 2002 ▸), anti-inflammatory (Thakurdesai *et al.*, 2007 ▸), anti­hypertensive (Serafin *et al.*, 1989 ▸) and anti-trichinellosis (Mavrova *et al.*, 2007 ▸) activities. In addition, they are considered to be important moieties for the development of mol­ecules of pharmaceutical inter­est (Mondieig *et al.*, 2013 ▸; Lakhrissi *et al.*, 2008 ▸). As a continuation of our research devoted to the study of the cyclo­addition reactions involving benzimidazolone derivatives (Sebbar *et al.*, 2016 ▸; Saber *et al.*, 2020 ▸), we report herein the synthesis, the mol­ecular and crystal structures of the title compound along with the results of the Hirshfeld surface analysis and the density functional theory (DFT) computational calculations carried out at the B3LYP/6–311 G(d,p) level in order to compare the theoretical and experimentally determined mol­ecular structures in the solid state.
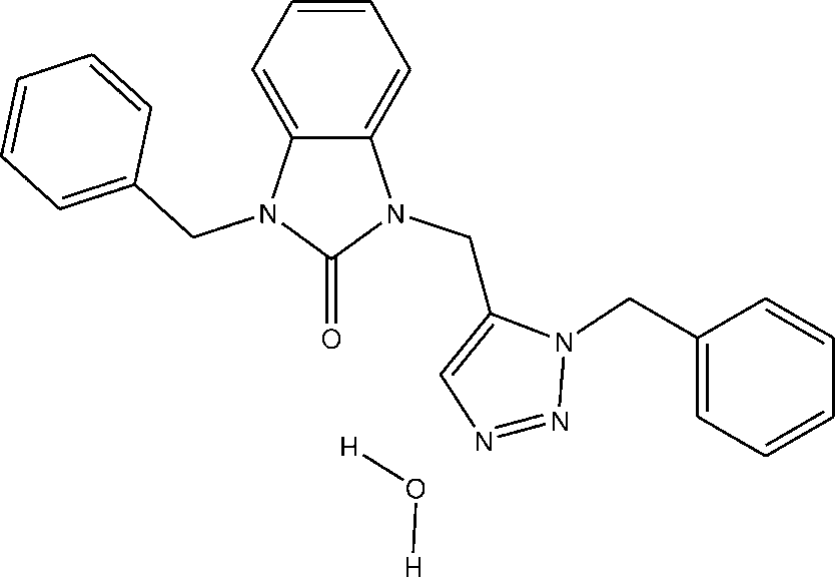


## Structural commentary   

**Figure 1:**
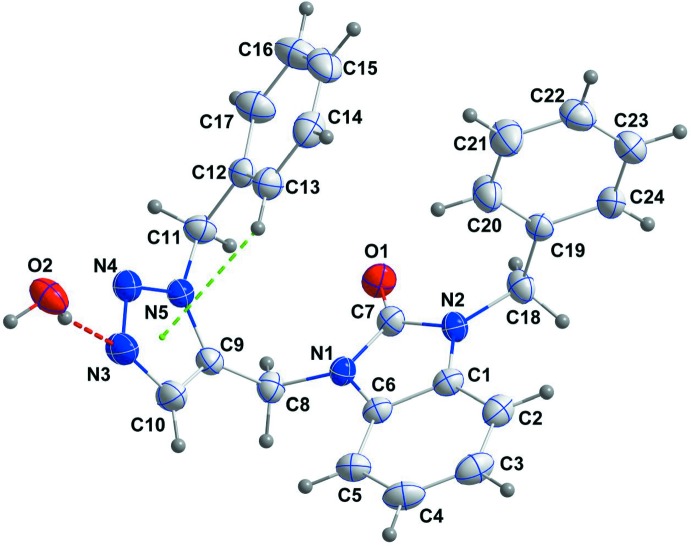
The mol­ecular structure of the title compound with the atom-numbering scheme. Displacement ellipsoids are drawn at the 50% probability level. The O—H_UncoordW_⋯N_Trz_ (UncoordW = uncoordinated water, Trz = triazole) hydrogen bond is shown by a red dashed line while the intra­molecular C—H⋯π(ring) inter­action is depicted by a green dashed line.

The title mol­ecule, (I), adopts a U-shaped conformation with an H20⋯C14 separation of 2.83 Å, which is very close to a normal van der Waals contact (2.90 Å). The orientation of the C11–C17 benzyl group is partly determined by an intra­molecular C13—H13⋯*Cg* inter­action, where *Cg* is the centroid of the triazole (C9/C10/N3–N5), ring *C* (Fig. 1 ▸ and Table 1 ▸). The di­hydro­benzo­diazole unit is not quite planar, as indicated by the dihedral angle of 2.50 (8)° between the constituent rings *A* (C1–C6) and *B* (N1/N2/C1/C6/C7) and the deviation of atom C7 by 0.0418 (14) Å out of the mean plane through the whole unit. The benzene ring *D* (C12–C17) is inclined to the triazole ring *C* by 78.91 (11)° while the latter ring is inclined to the *B* ring by 64.70 (11)°. The dihedral angle between the mean planes of the *B* and *E* (C19–C24) rings is 87.67 (8)°.

## Supra­molecular features   

**Figure 2:**
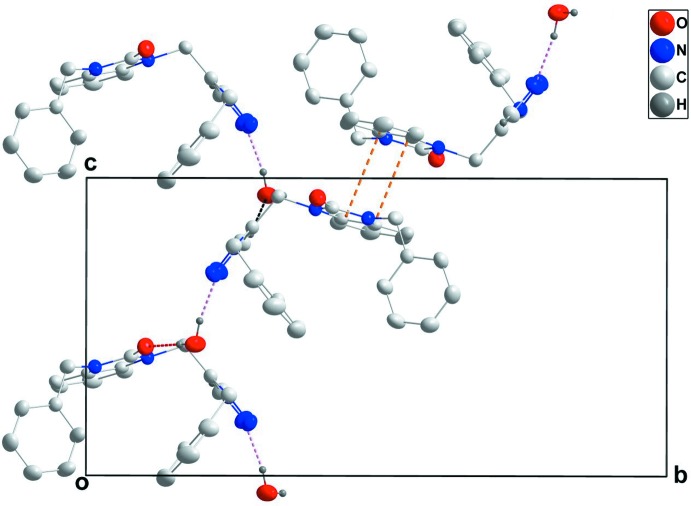
A partial packing diagram viewed along the *a*-axis direction with O—H_UncoordW_⋯O_Dhyr_, O—H_UncoordW_⋯N_Trz_ and C—H_Trz_⋯O_UncoordW_ (UncoordW = uncoordinated water, Dhyr = di­hydro, Trz = triazole) hydrogen bonds shown, respectively, as red, pink and black dashed lines. The π-stacking inter­actions are shown as orange dashed lines.

**Figure 3:**
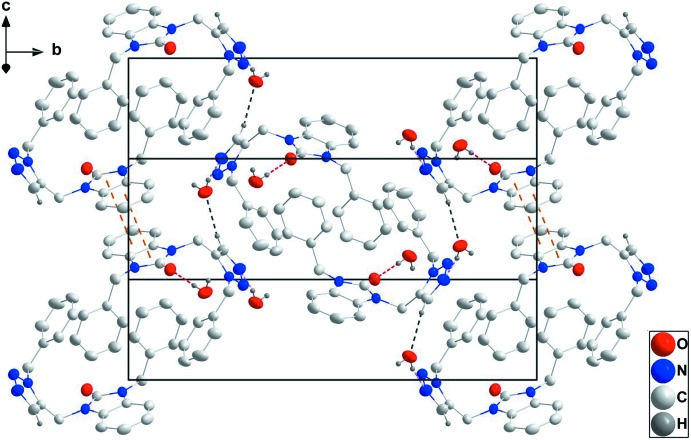
A partial packing diagram projected onto (301) with inter­molecular inter­actions depicted as in Fig. 2 ▸.

In the crystal, the mol­ecules form chains with the water mol­ecule of crystallization, which extend along [201] through O—H_UncoordW_⋯O_Dhyr_ and O—H_UncoordW_⋯N_Trz_ (UncoordW = uncoordinated water, Dhyr = di­hydro, Trz = triazole) hydrogen bonds (Table 1 ▸ and Fig. 2 ▸). The chains are connected into layers parallel to (010) by C—H_Trz_⋯O_UncoordW_ hydrogen bonds (Table 1 ▸ and Fig. 2 ▸). Inter­calation of the di­hydro­benzo­diazole groups between adjacent layers with concomitant head-to-tail π-stacking inter­actions between them [*Cg*2⋯*Cg*1^i^ = 3.5694 (11) Å where *Cg*1 and *Cg*2 are the centroids of the *A* and *B* rings, respectively; symmetry code: (i) −*x* + 1, −*y* + 1, −*z* + 2; dihedral angle = 2.50 (10)°] leads to the final three-dimensional structure (Fig. 3 ▸).

## Hirshfeld surface analysis   

**Figure 4:**
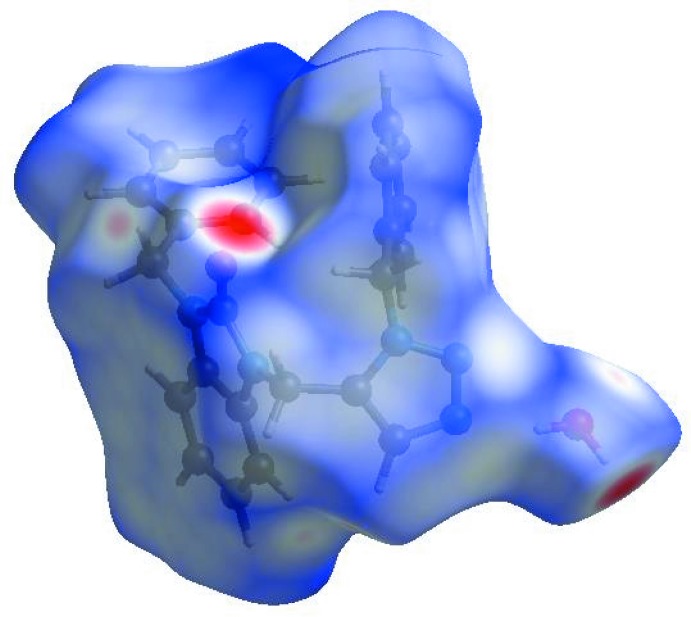
View of the three-dimensional Hirshfeld surface of the title compound plotted over *d*_norm_ in the range −0.5603 to 1.3285 a.u.

**Figure 5:**
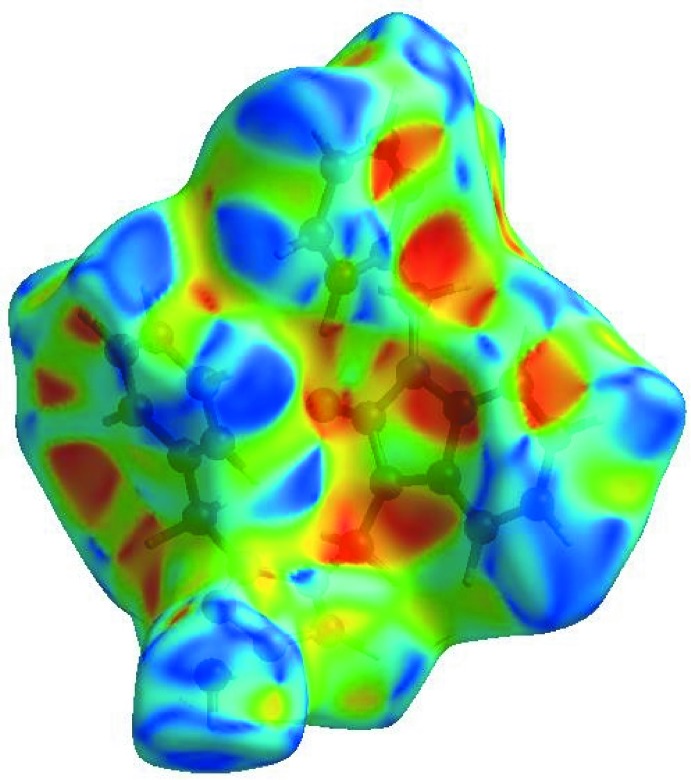
Hirshfeld surface of the title compound plotted over shape-index.

In order to visualize the inter­molecular inter­actions in the crystal of the title compound, a Hirshfeld surface (HS) analysis (Hirshfeld, 1977 ▸; Spackman & Jayatilaka, 2009 ▸) was carried out using *Crystal Explorer 17.5* (Turner *et al.*, 2017 ▸). In the HS plotted over *d*_norm_ (Fig. 4 ▸), white areas indicates contacts with distances equal to the sum of van der Waals radii, and the red and blue colours indicate distances shorter (in close contact) or longer (distinct contact), respectively, than the van der Waals radii (Venkatesan *et al.*, 2016 ▸). The bright-red spots appearing near O1 and hydrogen atom H2*B* indicate their roles as the respective donors and acceptors. The shape-index of the HS is a tool to visualize the π–π stacking by the presence of adjacent red and blue triangles; if there are no adjacent red and/or blue triangles, then there are no π–π inter­actions. Fig. 5 ▸ clearly suggests that there are π–π inter­actions in (I).

**Figure 6:**
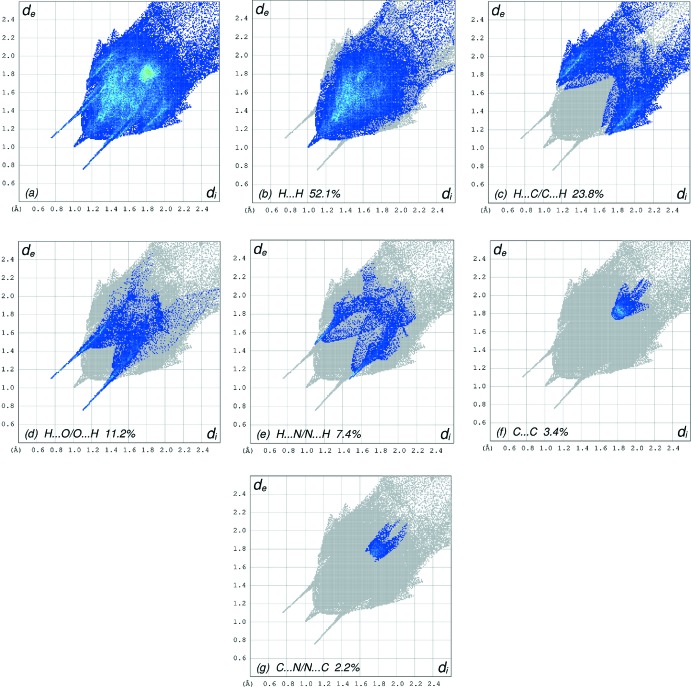
The full two-dimensional fingerprint plots for the title compound, showing (*a*) all inter­actions, and delineated into (*b*) H⋯H, (*c*) H⋯C/C⋯H, (*d*) H⋯O/O⋯H, (*e*) H⋯N/N⋯H, (*f*) C⋯C and (*g*) C⋯N/N⋯C inter­actions. The *d*_i_ and *d*_e_ values are the closest inter­nal and external distances (in Å) from given points on the Hirshfeld surface contacts.

The overall two-dimensional fingerprint plot, Fig. 6 ▸ *a*, and those delineated into H⋯H, H⋯C/C⋯H, H⋯O/O⋯H, H⋯N/N⋯H, C⋯C and C⋯N/N⋯C contacts (McKinnon *et al.*, 2007 ▸) are illustrated in Fig. 6 ▸ *b*–*g*, respectively, together with their relative contributions to the Hirshfeld surface. The most important inter­action (Table 2 ▸) is H⋯H, contributing 52.1% to the overall crystal packing, which is reflected in Fig. 6 ▸ *b* as widely scattered points of high density due to the large hydrogen content of the mol­ecule with the tip at *d*_e_ = *d*_i_ = 1.00 Å. The presence of C—H⋯π inter­actions give rise to pairs of characteristic wings in the fingerprint plot delineated into H⋯C/C⋯H contacts (23.8% contribution to the HS), Fig. 6 ▸ *c*,(Table 2 ▸) with triple pairs of spikes with the tips at *d*_e_ + *d*_i_ = 2.86, 2.82 and 2.85 Å. The scattered points in the pair of wings in the fingerprint plots delineated into H⋯O/O⋯H contacts (11.2% contribution), Fig. 6 ▸ *d*, have a symmetrical distribution with the edges at *d*_e_ + *d*_i_ = 1.85 Å. The H⋯N/N⋯N contacts, contributing 7.4% to the overall crystal packing, are shown in Fig. 6 ▸ *e* as widely scattered points with the tips at *d*_e_ + *d*_i_ = 2.56 Å. The C⋯C contacts, Fig. 6 ▸ *f*, have an arrow-shaped distribution of points with the tip at *d*_e_ = *d*_i_ = 1.77 Å. Finally, the C⋯N/N⋯C inter­actions (2.2%) are reflected in Fig. 6 ▸ *g* as tiny characteristic wings with the tips at *d*_e_ + *d*_i_ = 3.44 Å.

**Figure 7:**
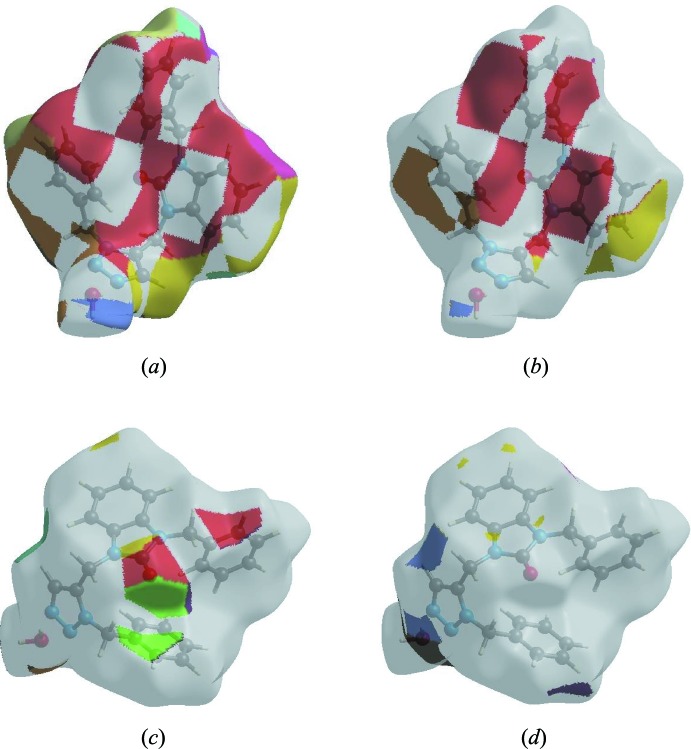
The Hirshfeld surface representations with the function *d*_norm_ plotted onto the surface for (*a*) H⋯H, (*b*) H⋯C/C⋯H, (*c*) H⋯O/O⋯H and (*d*) H⋯N/N⋯H inter­actions.

The Hirshfeld surface representations with the function *d*_norm_ plotted onto the surface are shown for the H⋯H, H⋯C/C⋯H, H⋯O/O⋯H and H⋯N/N⋯H inter­actions in Fig. 7 ▸ *a*–*d*, respectively.

The Hirshfeld surface analysis confirms the importance of H-atom contacts in establishing the packing. The large number of H⋯H, H⋯C/C⋯H and H⋯O/O⋯H inter­actions suggest that van der Waals inter­actions and hydrogen bonding play the major roles in the crystal packing (Hathwar *et al.*, 2015 ▸).

## Database survey   

An N-substituted benzoimidazol-2-one analogue (Saber *et al.*, 2018*a* ▸,*b* ▸; Saber *et al.*, 2020 ▸) and other similar compounds have also been reported (Belaziz *et al.*, 2012 ▸, 2013 ▸; Bouayad *et al.*, 2015 ▸). In derivatives of benzimidazolin-2-one in which both nitro­gen atoms form exocyclic C—N bonds, the bicyclic ring system is either planar, has a slight twist end-to-end, or, in the cases where the exocyclic substituents form a ring, has a very shallow bowl shape. The closest examples to the title compound are **2** (Saber *et al.*, 2018*a* ▸) and **3** (Saber *et al.*, 2018*b* ▸) with **4** (Díez-Barra *et al.*, 1997 ▸) as a more distant relative. In **3** [Chem scheme2], the C—N bond, connecting the nitro­gen atoms to form exocyclic units are 1.4632 (15) and 1.4525 (16) Å, while in the title compound, the C—N bonds are 1.4301 (15) and 1.4525 (16) Å. In the bicyclic units, they are in an *anti*-arrangement, and this is basically the same for **2** [Chem scheme2]. Inter­estingly, the three bicyclic units in **4** [Chem scheme2] are close to all being *syn* to one another.
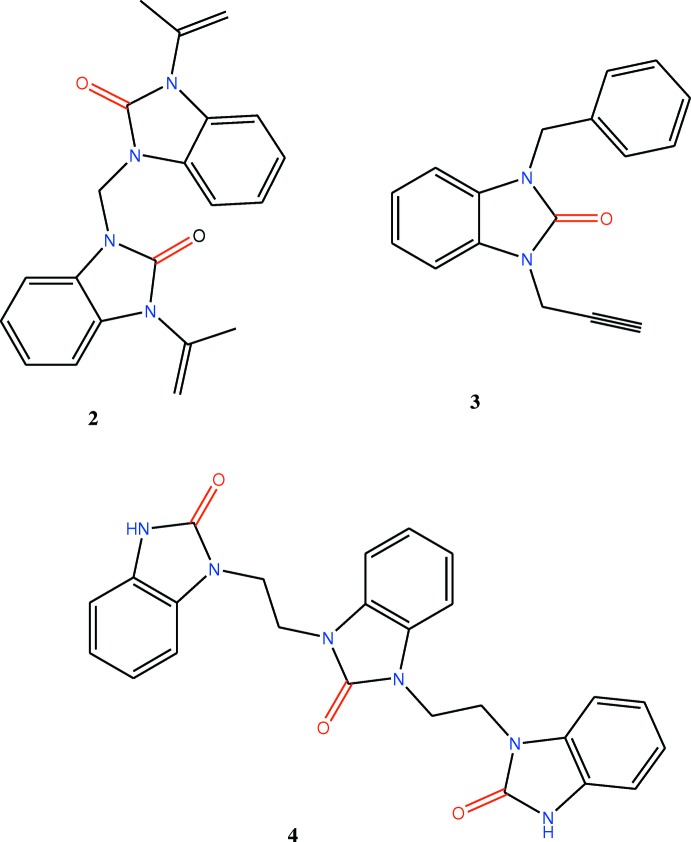


## DFT calculations   

**Figure 8:**
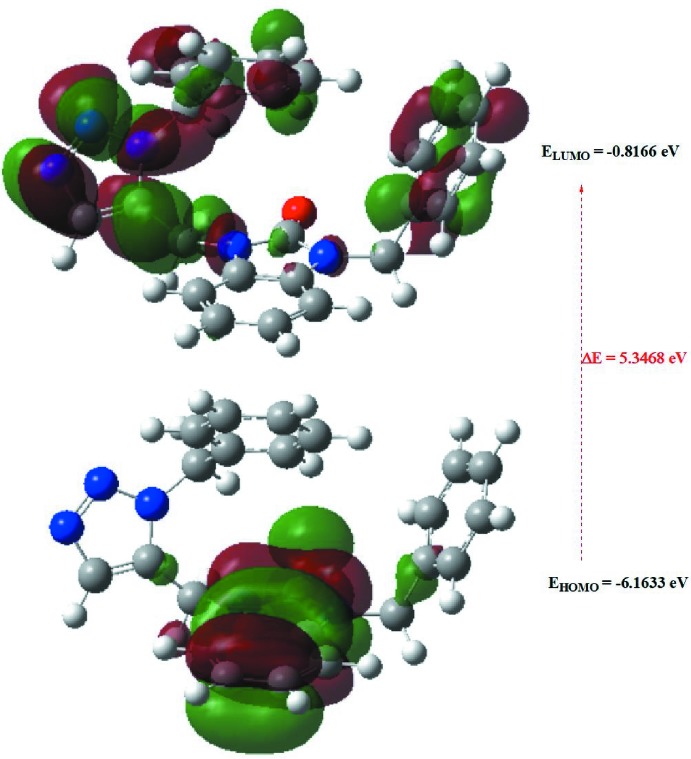
The energy band gap of the title compound, (I).

The optimized structure of the title compound, (I), in the gas phase was generated theoretically *via* density functional theory (DFT) using standard B3LYP functional and 6–311 G(d,p) basis-set calculations (Becke, 1993 ▸) as implemented in *GAUSSIAN 09* (Frisch *et al.*, 2009 ▸). The theoretical and experimental results are in good agreement (Table 3 ▸). The highest-occupied mol­ecular orbital (HOMO), acting as an electron donor, and the lowest-unoccupied mol­ecular orbital (LUMO), acting as an electron acceptor, are very important parameters for quantum chemistry. When the energy gap is small, the mol­ecule is highly polarizable and has high chemical reactivity. The DFT calculations provide some important information on the reactivity and site selectivity of the mol­ecular framework. *E*_HOMO_ and *E*_LUMO_ clarify the inevitable charge-exchange collaboration inside the studied material, electronegativity (χ), hardness (η), potential (μ), electrophilicity (ω) and softness (σ) are recorded in Table 4 ▸. The significance of η and σ is to evaluate both the reactivity and stability. The electron transition from the HOMO to the LUMO energy level is shown in Fig. 8 ▸. The HOMO and LUMO are localized in the plane extending over the whole 1-benzyl-3-[(1-benzyl-1*H*-1,2,3-triazol-4-yl)meth­yl]-2,3-di­hydro-1*H*-1,3-benzo­diazol-2-one hydrate ring. The energy band gap [Δ*E* = *E*_LUMO_ - *E*_HOMO_] of the mol­ecule is 5.3468 eV, and the frontier mol­ecular orbital energies, *E*_HOMO_ and *E*_LUMO_ are −6.1633 and −0.8166 eV, respectively.

## Synthesis and crystallization   

To a mixture of 3-methyl-1-(prop-2-yn­yl)-3,4-di­hydro­quinoxalin-2(1H)-one (0.65 mmol) in ethanol (20 ml) was added 1-(azido­meth­yl)benzene (1.04 mmol). The mixture was stirred under reflux for 24 h. After completion of the reaction (monitored by TLC), the solution was concentrated and the residue obtained was purified by column chromatography on silica gel by using as eluent a mixture (hexa­ne/ethyl acetate: 9/1). The isolated solid product was recrystallized from ethanol to afford yellow crystals (yield: in 19%).

## Refinement   

The experimental details including the crystal data, data collection and refinement are summarized in Table 5 ▸. Hydrogen atoms were included as riding contributions in idealized positions with C—H = 0.95–0.99 Å and *U*_iso_(H) = 1.2*U*_eq_(C).

## Supplementary Material

Crystal structure: contains datablock(s) I, global. DOI: 10.1107/S2056989019016876/lh5940sup1.cif

Structure factors: contains datablock(s) I. DOI: 10.1107/S2056989019016876/lh5940Isup2.hkl

Supporting information file. DOI: 10.1107/S2056989019016876/lh5940Isup3.cdx

Supporting information file. DOI: 10.1107/S2056989019016876/lh5940Isup4.cml

CCDC reference: 1972575

Additional supporting information:  crystallographic information; 3D view; checkCIF report

## Figures and Tables

**Table 1:** Hydrogen-bond geometry (Å, °) *Cg* is the centroid of the triazole ring *C* (C9/C10/N3–N5).

*D*—H⋯*A*	*D*—H	H⋯*A*	*D*⋯*A*	*D*—H⋯*A*
O2—H2*A*⋯N3	0.87	2.04	2.892 (2)	166
O2—H2*B*⋯O1^i^	0.87	2.00	2.865 (2)	176
C10—H10⋯O2^v^	0.95	2.48	3.402 (3)	164
C13—H13⋯*Cg*	0.95	2.83	3.451 (3)	124

Symmetry codes: (i) 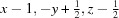; (v) 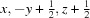.

**Table 2:** Selected interatomic distances (Å)

O2⋯O1^i^	2.865 (2)	C10⋯H5	2.98
O2⋯C17^i^	3.192 (3)	C11⋯H8*B*	2.90
O2⋯N3	2.892 (2)	C14⋯H20	2.83
O1⋯H8*B*	2.55	C18⋯H2	2.98
O1⋯H11*B*	2.81	C22⋯H16^vii^	2.98
O1⋯H18*A*	2.56	C22⋯H13^vi^	2.97
O1⋯H18*A* ^ii^	2.87	C23⋯H16^vii^	2.97
O2⋯H5^iii^	2.64	H2⋯N4^viii^	2.78
O2⋯H11*B* ^i^	2.77	H2*A*⋯N4	2.62
O2⋯H17^i^	2.71	H2*A*⋯N3	2.04
N4⋯C13	3.200 (3)	H2*B*⋯O1^i^	2.00
N2⋯H20	2.60	H2*B*⋯H11*B* ^i^	2.48
N4⋯H13	2.73	H3⋯H15^vi^	2.48
N5⋯H13	2.52	H4⋯H18*A* ^ix^	2.57
C1⋯C20	3.557 (3)	H5⋯H10	2.44
C2⋯C6^iv^	3.542 (3)	H8*A*⋯N4^v^	2.67
C3⋯C7^iv^	3.540 (3)	H8*B*⋯H11*B*	2.27
C5⋯C9	3.592 (3)	H10⋯O2^v^	2.48
C9⋯C5	3.592 (3)	H10⋯H17^ix^	2.46
C10⋯O2^v^	3.402 (3)	H11*A*⋯C15^v^	2.92
C11⋯C15^v^	3.421 (3)	H11*A*⋯H17	2.51
C14⋯C20	3.505 (3)	H16⋯H23^vii^	2.44
C2⋯H18*B*	2.98	H16⋯H22^vii^	2.46
C3⋯H15^vi^	2.88	H18*A*⋯H18*A* ^ii^	2.19
C8⋯H11*B*	2.79	H18*B*⋯H24	2.43
C8⋯H5	2.99	H24⋯N3^viii^	2.76

Symmetry codes: (i) 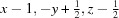; (ii) 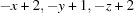; (iii) 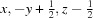; (iv) 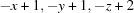; (v) 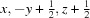; (vi) 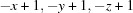; (vii) 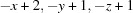; (viii) 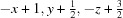; (ix) 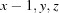.

**Table 3:** Comparison of selected (X-ray and DFT) geometric data (Å, °)

Bonds/angles	X-ray	B3LYP/6–311G(d,p)
O1—C7	1.225 (2)	1.25497
N1—C7	1.384 (2)	1.40076
N1—C6	1.397 (2)	1.40603
N1—C8	1.452 (2)	1.46502
N2—C7	1.379 (2)	1.39180
N2—C1	1.395 (2)	1.40574
N2—C18	1.450 (2)	1.47028
N3—N4	1.314 (2)	1.32954
N3—C10	1.358 (3)	1.37406
N4—N5	1.347 (2)	1.38781
N5—C9	1.356 (2)	1.37548
N5—C11	1.452 (2)	1.47090
C7—N1—C6	109.72 (15)	109.64541
C7—N1—C8	123.68 (15)	122.59694
C6—N1—C8	125.96 (15)	127.83740
C7—N2—C1	109.90 (15)	109.86320
C7—N2—C18	123.91 (16)	122.77835
C1—N2—C18	125.82 (16)	128.23580
N4—N3—C10	108.55 (17)	108.75382
N3—N4—N5	107.17 (16)	107.07997
N4—N5—C9	111.14 (15)	110.25168
N4—N5—C11	118.44 (16)	118.90455

**Table 4:** Calculated energies

Mol­ecular Energy (a.u.) (eV)	Compound (I)
Total Energy, *TE* (eV)	−34723.0011
*E*_HOMO_ (eV)	−6.1633
*E*_LUMO_ (eV)	−0.8166
Gap *ΔE* (eV)	5.3468
Dipole moment, μ (Debye)	5.5500
Ionization potential *I* (eV)	6.1633
Electron affinity, *A*	0.8166
Electronegativity, χ	3.4900
Hardness, η	2.6734
Electrophilicity index, ω	2.2780
Softness, σ	0.3741
Fraction of electron transferred, *ΔN*	0.6565

**Table 5:** Experimental details

Crystal data	
Chemical formula	C_24_H_21_N_5_O·H_2_O
*M*_r_	413.47
Crystal system, space group	Monoclinic, *P*2_1_/*c*
Temperature (K)	150
*a*, *b*, *c* (Å)	9.0872 (2), 21.1012 (4), 11.7134 (2)
β (°)	112.654 (1)
*V* (Å^3^)	2072.77 (7)
*Z*	4
Radiation type	Cu *K*α
μ (mm^−1^)	0.70
Crystal size (mm)	0.18 × 0.08 × 0.01

Data collection	
Diffractometer	Bruker D8 VENTURE PHOTON 100 CMOS
Absorption correction	Multi-scan (*SADABS*; Krause *et al.*, 2015 ▸)
*T*_min_, *T*_max_	0.85, 0.99
No. of measured, independent and observed [*I* > 2σ(*I*)] reflections	15080, 3887, 2909
*R*_int_	0.057
(sin θ/λ)_max_ (Å^−1^)	0.610

Refinement	
*R*[*F* ^2^ > 2σ(*F* ^2^)], *wR*(*F* ^2^), *S*	0.048, 0.113, 1.06
No. of reflections	3887
No. of parameters	280
H-atom treatment	H-atom parameters constrained
Δρ_max_, Δρ_min_ (e Å^−3^)	0.22, −0.22

Computer programs: *APEX3* and *SAINT* (Bruker, 2016 ▸), *SHELXT2014* (Sheldrick, 2015*a* ▸), *SHELXL2018* (Sheldrick, 2015*b* ▸) and *DIAMOND* (Brandenburg & Putz, 2012 ▸).

## supplementary crystallographic information

### Crystal data

**Table:**

C_24_H_21_N_5_O·H_2_O	*F*(000) = 872
*M**_r_* = 413.47	*D*_x_ = 1.325 Mg m^−^^3^
Monoclinic, *P*2_1_/*c*	Cu *K*α radiation, λ = 1.54178 Å
*a* = 9.0872 (2) Å	Cell parameters from 9060 reflections
*b* = 21.1012 (4) Å	θ = 4.2–70.2°
*c* = 11.7134 (2) Å	µ = 0.70 mm^−^^1^
β = 112.654 (1)°	*T* = 150 K
*V* = 2072.77 (7) Å^3^	Plate, colourless
*Z* = 4	0.18 × 0.08 × 0.01 mm

### Data collection

**Table:**

Bruker D8 VENTURE PHOTON 100 CMOS diffractometer	3887 independent reflections
Radiation source: INCOATEC IµS micro–focus source	2909 reflections with *I* > 2σ(*I*)
Mirror monochromator	*R*_int_ = 0.057
Detector resolution: 10.4167 pixels mm^-1^	θ_max_ = 70.2°, θ_min_ = 4.2°
ω scans	*h* = −11→10
Absorption correction: multi-scan (*SADABS*; Krause *et al.*, 2015)	*k* = −24→25
*T*_min_ = 0.85, *T*_max_ = 0.99	*l* = −14→13
15080 measured reflections	

### Refinement

**Table:**

Refinement on *F*^2^	Primary atom site location: structure-invariant direct methods
Least-squares matrix: full	Secondary atom site location: difference Fourier map
*R*[*F*^2^ > 2σ(*F*^2^)] = 0.048	Hydrogen site location: mixed
*wR*(*F*^2^) = 0.113	H-atom parameters constrained
*S* = 1.06	*w* = 1/[σ^2^(*F*_o_^2^) + (0.0367*P*)^2^ + 0.901*P*] where *P* = (*F*_o_^2^ + 2*F*_c_^2^)/3
3887 reflections	(Δ/σ)_max_ < 0.001
280 parameters	Δρ_max_ = 0.22 e Å^−^^3^
0 restraints	Δρ_min_ = −0.21 e Å^−^^3^

### Special details

**Table:**

Geometry. All esds (except the esd in the dihedral angle between two l.s. planes) are estimated using the full covariance matrix. The cell esds are taken into account individually in the estimation of esds in distances, angles and torsion angles; correlations between esds in cell parameters are only used when they are defined by crystal symmetry. An approximate (isotropic) treatment of cell esds is used for estimating esds involving l.s. planes.
Refinement. Refinement of F^2^ against ALL reflections. The weighted R-factor wR and goodness of fit S are based on F^2^, conventional R-factors R are based on F, with F set to zero for negative F^2^. The threshold expression of F^2^ > 2sigma(F^2^) is used only for calculating R-factors(gt) etc. and is not relevant to the choice of reflections for refinement. R-factors based on F^2^ are statistically about twice as large as those based on F, and R- factors based on ALL data will be even larger. H-atoms attached to carbon were placed in calculated positions (C—H = 0.95 - 0.99 Å) while those attached to oxygen were placed in locations derived from a difference map and their coordinates adjusted to give O—H = 0.87 Å. All were included as riding contributions with isotropic displacement parameters 1.2 - 1.5 times those of the attached atoms.

### Fractional atomic coordinates and isotropic or equivalent isotropic displacement parameters (Å^2^)

**Table:**

	*x*	*y*	*z*	*U*_iso_*/*U*_eq_	
O1	0.85567 (15)	0.39779 (7)	0.93327 (13)	0.0375 (4)	
N1	0.58475 (17)	0.39409 (7)	0.89638 (14)	0.0267 (3)	
N2	0.68572 (18)	0.48484 (7)	0.86568 (14)	0.0278 (3)	
N3	0.3094 (2)	0.22818 (9)	0.68794 (17)	0.0426 (5)	
N4	0.4467 (2)	0.21861 (8)	0.67770 (16)	0.0378 (4)	
N5	0.55663 (19)	0.25352 (8)	0.76599 (15)	0.0300 (4)	
C1	0.5258 (2)	0.49606 (9)	0.84381 (16)	0.0274 (4)	
C2	0.4364 (2)	0.55101 (10)	0.81370 (18)	0.0350 (5)	
H2	0.480398	0.590041	0.801540	0.042*	
C3	0.2785 (3)	0.54622 (11)	0.80211 (19)	0.0411 (5)	
H3	0.213195	0.583008	0.781973	0.049*	
C4	0.2141 (2)	0.48927 (12)	0.8192 (2)	0.0416 (5)	
H4	0.105243	0.487817	0.808961	0.050*	
C5	0.3051 (2)	0.43389 (11)	0.85099 (18)	0.0342 (5)	
H5	0.261424	0.394902	0.863712	0.041*	
C6	0.4618 (2)	0.43874 (9)	0.86300 (16)	0.0263 (4)	
C7	0.7242 (2)	0.42276 (9)	0.90173 (17)	0.0275 (4)	
C8	0.5772 (2)	0.32986 (9)	0.93828 (18)	0.0307 (4)	
H8A	0.523147	0.330364	0.997257	0.037*	
H8B	0.686995	0.313896	0.982830	0.037*	
C9	0.4903 (2)	0.28571 (9)	0.83436 (17)	0.0288 (4)	
C10	0.3330 (2)	0.26854 (10)	0.7837 (2)	0.0381 (5)	
H10	0.252957	0.282630	0.811211	0.046*	
C11	0.7188 (2)	0.25400 (10)	0.77111 (19)	0.0337 (5)	
H11A	0.752881	0.209810	0.766704	0.040*	
H11B	0.790188	0.271831	0.851613	0.040*	
C12	0.7378 (2)	0.29187 (9)	0.66852 (17)	0.0303 (4)	
C13	0.6180 (3)	0.32884 (10)	0.58683 (19)	0.0372 (5)	
H13	0.516342	0.330255	0.592204	0.045*	
C14	0.6458 (3)	0.36398 (11)	0.4968 (2)	0.0439 (5)	
H14	0.562951	0.389101	0.440157	0.053*	
C15	0.7931 (3)	0.36233 (12)	0.4897 (2)	0.0499 (6)	
H15	0.813571	0.387548	0.430265	0.060*	
C16	0.9119 (3)	0.32390 (14)	0.5694 (2)	0.0531 (7)	
H16	1.012885	0.321923	0.563017	0.064*	
C17	0.8840 (3)	0.28853 (12)	0.6578 (2)	0.0439 (6)	
H17	0.965324	0.261779	0.711555	0.053*	
C18	0.8011 (2)	0.53227 (10)	0.86554 (18)	0.0336 (5)	
H18A	0.909556	0.515338	0.911413	0.040*	
H18B	0.787800	0.570113	0.910627	0.040*	
C19	0.7883 (2)	0.55247 (9)	0.73841 (17)	0.0280 (4)	
C20	0.7223 (3)	0.51411 (11)	0.6354 (2)	0.0435 (5)	
H20	0.679963	0.473924	0.643246	0.052*	
C21	0.7171 (3)	0.53360 (12)	0.5208 (2)	0.0493 (6)	
H21	0.671787	0.506684	0.450937	0.059*	
C22	0.7773 (3)	0.59167 (12)	0.5081 (2)	0.0430 (5)	
H22	0.772714	0.605182	0.429394	0.052*	
C23	0.8446 (3)	0.63033 (11)	0.6103 (2)	0.0432 (5)	
H23	0.886487	0.670536	0.602007	0.052*	
C24	0.8509 (2)	0.61052 (10)	0.7246 (2)	0.0357 (5)	
H24	0.898875	0.637078	0.794666	0.043*	
O2	0.10404 (17)	0.18802 (8)	0.44186 (14)	0.0478 (4)	
H2A	0.155922	0.195519	0.520249	0.072*	
H2B	0.031707	0.160858	0.441258	0.072*	

### Atomic displacement parameters (Å^2^)

**Table:**

	*U*^11^	*U*^22^	*U*^33^	*U*^12^	*U*^13^	*U*^23^
O1	0.0242 (7)	0.0395 (9)	0.0441 (8)	0.0055 (6)	0.0078 (6)	−0.0002 (7)
N1	0.0247 (8)	0.0244 (8)	0.0286 (8)	0.0013 (6)	0.0077 (6)	−0.0006 (6)
N2	0.0259 (8)	0.0263 (9)	0.0283 (8)	−0.0016 (7)	0.0072 (6)	0.0013 (7)
N3	0.0363 (9)	0.0486 (12)	0.0454 (11)	−0.0144 (9)	0.0184 (8)	−0.0126 (9)
N4	0.0385 (9)	0.0358 (10)	0.0409 (10)	−0.0099 (8)	0.0174 (8)	−0.0081 (8)
N5	0.0306 (8)	0.0291 (9)	0.0316 (9)	−0.0037 (7)	0.0132 (7)	−0.0018 (7)
C1	0.0267 (9)	0.0319 (11)	0.0206 (9)	0.0029 (8)	0.0059 (7)	−0.0007 (7)
C2	0.0430 (11)	0.0328 (12)	0.0277 (10)	0.0085 (9)	0.0120 (9)	0.0036 (8)
C3	0.0438 (12)	0.0442 (14)	0.0339 (11)	0.0195 (10)	0.0134 (9)	0.0054 (9)
C4	0.0303 (10)	0.0591 (15)	0.0360 (12)	0.0118 (10)	0.0132 (9)	0.0009 (10)
C5	0.0302 (10)	0.0429 (13)	0.0311 (10)	0.0015 (9)	0.0135 (8)	−0.0007 (9)
C6	0.0255 (9)	0.0303 (11)	0.0215 (9)	0.0024 (8)	0.0071 (7)	−0.0011 (8)
C7	0.0232 (9)	0.0302 (10)	0.0252 (9)	−0.0006 (8)	0.0050 (7)	−0.0023 (8)
C8	0.0345 (10)	0.0279 (11)	0.0280 (10)	0.0011 (8)	0.0101 (8)	0.0026 (8)
C9	0.0309 (9)	0.0272 (10)	0.0297 (10)	−0.0018 (8)	0.0132 (8)	0.0019 (8)
C10	0.0350 (11)	0.0409 (13)	0.0416 (12)	−0.0073 (9)	0.0182 (9)	−0.0083 (10)
C11	0.0288 (10)	0.0375 (12)	0.0362 (11)	0.0025 (9)	0.0138 (8)	0.0018 (9)
C12	0.0303 (10)	0.0318 (11)	0.0283 (10)	−0.0052 (8)	0.0108 (8)	−0.0047 (8)
C13	0.0401 (11)	0.0333 (12)	0.0401 (12)	0.0026 (9)	0.0177 (9)	0.0022 (9)
C14	0.0584 (14)	0.0363 (13)	0.0350 (12)	0.0035 (11)	0.0159 (10)	0.0016 (9)
C15	0.0679 (16)	0.0510 (15)	0.0377 (13)	−0.0150 (13)	0.0279 (12)	−0.0003 (11)
C16	0.0404 (12)	0.0785 (19)	0.0447 (14)	−0.0136 (13)	0.0211 (11)	0.0002 (13)
C17	0.0313 (10)	0.0601 (16)	0.0386 (12)	−0.0021 (10)	0.0115 (9)	0.0025 (11)
C18	0.0328 (10)	0.0340 (11)	0.0296 (10)	−0.0092 (9)	0.0070 (8)	−0.0023 (9)
C19	0.0240 (9)	0.0285 (10)	0.0309 (10)	−0.0005 (8)	0.0099 (7)	−0.0008 (8)
C20	0.0551 (14)	0.0388 (13)	0.0352 (12)	−0.0153 (11)	0.0159 (10)	−0.0061 (10)
C21	0.0625 (15)	0.0503 (15)	0.0340 (12)	−0.0167 (12)	0.0173 (11)	−0.0101 (11)
C22	0.0454 (12)	0.0512 (15)	0.0351 (12)	−0.0047 (11)	0.0188 (10)	0.0023 (10)
C23	0.0494 (13)	0.0384 (13)	0.0491 (13)	−0.0075 (10)	0.0272 (11)	0.0008 (10)
C24	0.0376 (11)	0.0323 (11)	0.0393 (12)	−0.0052 (9)	0.0171 (9)	−0.0062 (9)
O2	0.0397 (8)	0.0618 (11)	0.0395 (9)	−0.0154 (8)	0.0128 (7)	0.0018 (8)

### Geometric parameters (Å, º)

**Table:**

O1—C7	1.225 (2)	C11—H11B	0.9900
N1—C7	1.384 (2)	C12—C13	1.381 (3)
N1—C6	1.397 (2)	C12—C17	1.384 (3)
N1—C8	1.452 (2)	C13—C14	1.390 (3)
N2—C7	1.379 (2)	C13—H13	0.9500
N2—C1	1.395 (2)	C14—C15	1.373 (3)
N2—C18	1.450 (2)	C14—H14	0.9500
N3—N4	1.314 (2)	C15—C16	1.385 (4)
N3—C10	1.358 (3)	C15—H15	0.9500
N4—N5	1.347 (2)	C16—C17	1.377 (3)
N5—C9	1.356 (2)	C16—H16	0.9500
N5—C11	1.452 (2)	C17—H17	0.9500
C1—C2	1.381 (3)	C18—C19	1.510 (3)
C1—C6	1.397 (3)	C18—H18A	0.9900
C2—C3	1.392 (3)	C18—H18B	0.9900
C2—H2	0.9500	C19—C20	1.383 (3)
C3—C4	1.384 (3)	C19—C24	1.386 (3)
C3—H3	0.9500	C20—C21	1.387 (3)
C4—C5	1.397 (3)	C20—H20	0.9500
C4—H4	0.9500	C21—C22	1.373 (3)
C5—C6	1.379 (3)	C21—H21	0.9500
C5—H5	0.9500	C22—C23	1.382 (3)
C8—C9	1.494 (3)	C22—H22	0.9500
C8—H8A	0.9900	C23—C24	1.383 (3)
C8—H8B	0.9900	C23—H23	0.9500
C9—C10	1.368 (3)	C24—H24	0.9500
C10—H10	0.9500	O2—H2A	0.8700
C11—C12	1.507 (3)	O2—H2B	0.8701
C11—H11A	0.9900		
			
O2···O1^i^	2.865 (2)	C10···H5	2.98
O2···C17^i^	3.192 (3)	C11···H8B	2.90
O2···N3	2.892 (2)	C14···H20	2.83
O1···H8B	2.55	C18···H2	2.98
O1···H11B	2.81	C22···H16^vii^	2.98
O1···H18A	2.56	C22···H13^vi^	2.97
O1···H18A^ii^	2.87	C23···H16^vii^	2.97
O2···H5^iii^	2.64	H2···N4^viii^	2.78
O2···H11B^i^	2.77	H2A···N4	2.62
O2···H17^i^	2.71	H2A···N3	2.04
N4···C13	3.200 (3)	H2B···O1^i^	2.00
N2···H20	2.60	H2B···H11B^i^	2.48
N4···H13	2.73	H3···H15^vi^	2.48
N5···H13	2.52	H4···H18A^ix^	2.57
C1···C20	3.557 (3)	H5···H10	2.44
C2···C6^iv^	3.542 (3)	H8A···N4^v^	2.67
C3···C7^iv^	3.540 (3)	H8B···H11B	2.27
C5···C9	3.592 (3)	H10···O2^v^	2.48
C9···C5	3.592 (3)	H10···H17^ix^	2.46
C10···O2^v^	3.402 (3)	H11A···C15^v^	2.92
C11···C15^v^	3.421 (3)	H11A···H17	2.51
C14···C20	3.505 (3)	H16···H23^vii^	2.44
C2···H18B	2.98	H16···H22^vii^	2.46
C3···H15^vi^	2.88	H18A···H18A^ii^	2.19
C8···H11B	2.79	H18B···H24	2.43
C8···H5	2.99	H24···N3^viii^	2.76
			
C7—N1—C6	109.72 (15)	N5—C11—H11B	108.9
C7—N1—C8	123.68 (15)	C12—C11—H11B	108.9
C6—N1—C8	125.96 (15)	H11A—C11—H11B	107.7
C7—N2—C1	109.90 (15)	C13—C12—C17	119.63 (19)
C7—N2—C18	123.91 (16)	C13—C12—C11	123.39 (18)
C1—N2—C18	125.82 (16)	C17—C12—C11	116.99 (18)
N4—N3—C10	108.55 (17)	C12—C13—C14	120.1 (2)
N3—N4—N5	107.17 (16)	C12—C13—H13	119.9
N4—N5—C9	111.14 (15)	C14—C13—H13	119.9
N4—N5—C11	118.44 (16)	C15—C14—C13	119.9 (2)
C9—N5—C11	130.35 (17)	C15—C14—H14	120.1
C2—C1—N2	131.12 (19)	C13—C14—H14	120.1
C2—C1—C6	121.84 (18)	C14—C15—C16	120.0 (2)
N2—C1—C6	106.99 (16)	C14—C15—H15	120.0
C1—C2—C3	116.5 (2)	C16—C15—H15	120.0
C1—C2—H2	121.7	C17—C16—C15	120.2 (2)
C3—C2—H2	121.7	C17—C16—H16	119.9
C4—C3—C2	121.7 (2)	C15—C16—H16	119.9
C4—C3—H3	119.1	C16—C17—C12	120.1 (2)
C2—C3—H3	119.1	C16—C17—H17	119.9
C3—C4—C5	121.65 (19)	C12—C17—H17	119.9
C3—C4—H4	119.2	N2—C18—C19	114.52 (15)
C5—C4—H4	119.2	N2—C18—H18A	108.6
C6—C5—C4	116.6 (2)	C19—C18—H18A	108.6
C6—C5—H5	121.7	N2—C18—H18B	108.6
C4—C5—H5	121.7	C19—C18—H18B	108.6
C5—C6—C1	121.66 (18)	H18A—C18—H18B	107.6
C5—C6—N1	131.39 (18)	C20—C19—C24	118.34 (19)
C1—C6—N1	106.93 (15)	C20—C19—C18	122.54 (18)
O1—C7—N2	127.11 (18)	C24—C19—C18	119.06 (17)
O1—C7—N1	126.52 (18)	C19—C20—C21	120.8 (2)
N2—C7—N1	106.37 (15)	C19—C20—H20	119.6
N1—C8—C9	112.68 (15)	C21—C20—H20	119.6
N1—C8—H8A	109.1	C22—C21—C20	120.2 (2)
C9—C8—H8A	109.1	C22—C21—H21	119.9
N1—C8—H8B	109.1	C20—C21—H21	119.9
C9—C8—H8B	109.1	C21—C22—C23	119.6 (2)
H8A—C8—H8B	107.8	C21—C22—H22	120.2
N5—C9—C10	103.82 (17)	C23—C22—H22	120.2
N5—C9—C8	125.29 (17)	C22—C23—C24	120.0 (2)
C10—C9—C8	130.88 (18)	C22—C23—H23	120.0
N3—C10—C9	109.31 (18)	C24—C23—H23	120.0
N3—C10—H10	125.3	C23—C24—C19	121.0 (2)
C9—C10—H10	125.3	C23—C24—H24	119.5
N5—C11—C12	113.37 (16)	C19—C24—H24	119.5
N5—C11—H11A	108.9	H2A—O2—H2B	103.2
C12—C11—H11A	108.9		
			
C10—N3—N4—N5	−0.7 (2)	C11—N5—C9—C10	177.1 (2)
N3—N4—N5—C9	0.3 (2)	N4—N5—C9—C8	−178.77 (17)
N3—N4—N5—C11	−176.95 (17)	C11—N5—C9—C8	−1.9 (3)
C7—N2—C1—C2	175.59 (19)	N1—C8—C9—N5	86.6 (2)
C18—N2—C1—C2	2.4 (3)	N1—C8—C9—C10	−92.1 (3)
C7—N2—C1—C6	−1.9 (2)	N4—N3—C10—C9	0.9 (3)
C18—N2—C1—C6	−175.07 (17)	N5—C9—C10—N3	−0.7 (2)
N2—C1—C2—C3	−177.86 (19)	C8—C9—C10—N3	178.24 (19)
C6—C1—C2—C3	−0.7 (3)	N4—N5—C11—C12	73.3 (2)
C1—C2—C3—C4	−0.4 (3)	C9—N5—C11—C12	−103.4 (2)
C2—C3—C4—C5	1.1 (3)	N5—C11—C12—C13	7.5 (3)
C3—C4—C5—C6	−0.8 (3)	N5—C11—C12—C17	−172.44 (19)
C4—C5—C6—C1	−0.2 (3)	C17—C12—C13—C14	−1.9 (3)
C4—C5—C6—N1	178.04 (18)	C11—C12—C13—C14	178.1 (2)
C2—C1—C6—C5	1.0 (3)	C12—C13—C14—C15	−0.6 (3)
N2—C1—C6—C5	178.79 (17)	C13—C14—C15—C16	2.4 (4)
C2—C1—C6—N1	−177.65 (17)	C14—C15—C16—C17	−1.7 (4)
N2—C1—C6—N1	0.13 (19)	C15—C16—C17—C12	−0.8 (4)
C7—N1—C6—C5	−176.8 (2)	C13—C12—C17—C16	2.6 (3)
C8—N1—C6—C5	−5.8 (3)	C11—C12—C17—C16	−177.4 (2)
C7—N1—C6—C1	1.7 (2)	C7—N2—C18—C19	109.6 (2)
C8—N1—C6—C1	172.72 (16)	C1—N2—C18—C19	−78.2 (2)
C1—N2—C7—O1	−176.56 (19)	N2—C18—C19—C20	−24.7 (3)
C18—N2—C7—O1	−3.2 (3)	N2—C18—C19—C24	158.15 (18)
C1—N2—C7—N1	2.9 (2)	C24—C19—C20—C21	−0.8 (3)
C18—N2—C7—N1	176.23 (16)	C18—C19—C20—C21	−178.0 (2)
C6—N1—C7—O1	176.65 (18)	C19—C20—C21—C22	−0.2 (4)
C8—N1—C7—O1	5.4 (3)	C20—C21—C22—C23	0.7 (4)
C6—N1—C7—N2	−2.83 (19)	C21—C22—C23—C24	−0.1 (4)
C8—N1—C7—N2	−174.11 (16)	C22—C23—C24—C19	−1.0 (3)
C7—N1—C8—C9	−111.65 (19)	C20—C19—C24—C23	1.4 (3)
C6—N1—C8—C9	78.5 (2)	C18—C19—C24—C23	178.7 (2)
N4—N5—C9—C10	0.2 (2)		

Symmetry codes: (i) *x*−1, −*y*+1/2, *z*−1/2; (ii) −*x*+2, −*y*+1, −*z*+2; (iii) *x*, −*y*+1/2, *z*−1/2; (iv) −*x*+1, −*y*+1, −*z*+2; (v) *x*, −*y*+1/2, *z*+1/2; (vi) −*x*+1, −*y*+1, −*z*+1; (vii) −*x*+2, −*y*+1, −*z*+1; (viii) −*x*+1, *y*+1/2, −*z*+3/2; (ix) *x*−1, *y*, *z*.

### Hydrogen-bond geometry (Å, º)

*Cg* is the centroid of the triazole ring *C* (C9/C10/N3–N5).

**Table:**

*D*—H···*A*	*D*—H	H···*A*	*D*···*A*	*D*—H···*A*
O2—H2*A*···N3	0.87	2.04	2.892 (2)	166
O2—H2*B*···O1^i^	0.87	2.00	2.865 (2)	176
C10—H10···O2^v^	0.95	2.48	3.402 (3)	164
C13—H13···*Cg*	0.95	2.83	3.451 (3)	124

Symmetry codes: (i) *x*−1, −*y*+1/2, *z*−1/2; (v) *x*, −*y*+1/2, *z*+1/2.

## References

[bb1] Ayhan-Kılcıgil, G., Kus, G., Özdamar, E. D., Can-Eke, B. & Iscan, M. (2007). *Arch. Pharm. Chem. Life Sci.* **340**, 607–611. 10.1002/ardp.200700088 17994646

[bb2] Baxter, G. S. & Clarke, D. E. (1992). *Eur. J. Pharmacol.* **212**, 225–229. 10.1016/0014-2999(92)90333-y 1601064

[bb3] Becke, A. D. (1993). *J. Chem. Phys.* **98**, 5648–5652.

[bb4] Belaziz, D., Kandri Rodi, Y., Essassi, E. M. & El Ammari, L. (2012). *Acta Cryst.* E**68**, o1276. 10.1107/S1600536812013384 PMC3344429 22590191

[bb5] Belaziz, D., Kandri Rodi, Y., Ouazzani Chahdi, F., Essassi, E. M., Saadi, M. & El Ammari, L. (2013). *Acta Cryst.* E**69**, o122. 10.1107/S1600536812050726 PMC3588251 23476384

[bb6] Bouayad, K., Kandri Rodi, Y., Ouzidan, Y., Essassi, E. M., Saadi, M. & El Ammari, L. (2015). *Acta Cryst.* E**71**, o735–o736. 10.1107/S2056989015016102 PMC4647354 26594449

[bb7] Brandenburg, K. & Putz, H. (2012). *DIAMOND*. Crystal Impact GbR, Bonn, Germany.

[bb8] Bruker (2016). *APEX3* and *SAINT*, Bruker AXS, Inc., Madison, Wisconsin, USA.

[bb9] Díez-Barra, E., Dotor, J., de la Hoz, A., Foces-Foces, C., Enjalbal, C., Aubagnac, J. L., Claramunt, R. M. & Elguero, J. (1997). *Tetrahedron*, **53**, 7689–7704.

[bb10] Frisch, M. J., Trucks, G. W., Schlegel, H. B., Scuseria, G. E., Robb, M. A., Cheeseman, J. R., Scalmani, G., Barone, V., Mennucci, B., Petersson, G. A., Nakatsuji, H., Caricato, M., Li, X., Hratchian, H. P., Izmaylov, A. F., Bloino, J., Zheng, G., Sonnenberg, J. L., Hada, M., Ehara, M., Toyota, K., Fukuda, R., Hasegawa, J., Ishida, M., Nakajima, T., Honda, Y., Kitao, O., Nakai, H., Vreven, T., Montgomery, J. A., Jr., Peralta, J. E., Ogliaro, F., Bearpark, M., Heyd, J. J., Brothers, E., Kudin, K. N., Staroverov, V. N., Kobayashi, R., Normand, J., Raghavachari, K., Rendell, A., Burant, J. C., Iyengar, S. S., Tomasi, J., Cossi, M., Rega, N., Millam, J. M., Klene, M., Knox, J. E., Cross, J. B., Bakken, V., Adamo, C., Jaramillo, J., Gomperts, R., Stratmann, R. E., Yazyev, O., Austin, A. J., Cammi, R., Pomelli, C., Ochterski, J. W., Martin, R. L., Morokuma, K., Zakrzewski, V. G., Voth, G. A., Salvador, P., Dannenberg, J. J., Dapprich, S., Daniels, A. D., Farkas, ., Foresman, J. B., Ortiz, J. V., Cioslowski, J. & Fox, D. J. (2009). *GAUSSIAN 09*. Gaussian Inc., Wallingford, CT, US

[bb11] Gaba, M., Singh, S. & Mohan, C. (2014). *Eur. J. Med. Chem.* **76**, 494–505. 10.1016/j.ejmech.2014.01.030 24602792

[bb12] Garuti, L., Roberti, M., Malagoli, M., Rossi, T. & Castelli, M. (2000). *Bioorg. Med. Chem. Lett.* **10**, 2193–2195. 10.1016/s0960-894x(00)00429-7 11012027

[bb13] Hathwar, V. R., Sist, M., Jørgensen, M. R. V., Mamakhel, A. H., Wang, X., Hoffmann, C. M., Sugimoto, K., Overgaard, J. & Iversen, B. B. (2015). *IUCrJ*, **2**, 563–574. 10.1107/S2052252515012130 PMC4547824 26306198

[bb14] Hirshfeld, F. L. (1977). *Theor. Chim. Acta*, **44**, 129–138.

[bb15] Krause, L., Herbst-Irmer, R., Sheldrick, G. M. & Stalke, D. (2015). *J. Appl. Cryst.* **48**, 3–10. 10.1107/S1600576714022985 PMC4453166 26089746

[bb16] Lakhrissi, B., Benksim, A., Massoui, M., Essassi, el M., Lequart, V., Joly, N., Beaupère, D., Wadouachi, A. & Martin, P. (2008). *Carbohydr. Res.* **343**, 421–433. 10.1016/j.carres.2007.11.028 18155683

[bb17] Mavrova, A. Ts, Denkova, P., Tsenov, Y. A., Anichina, K. K. & Vutchev, D. I. (2007). *Bioorg. Med. Chem.* **15**, 6291–6297. 10.1016/j.bmc.2007.06.017 17600722

[bb18] McKinnon, J. J., Jayatilaka, D. & Spackman, M. A. (2007). *Chem. Commun.* pp. 3814–3816. 10.1039/b704980c 18217656

[bb19] Mondieig, D., Lakhrissi, L., El Assyry, A., Lakhrissi, B., Negrier, P., Essassi, E. M., Massoui, M., Michel Leger, J. & Benali, B. (2013). *J. Mar. Chim. Heterocycl.* **12**, 51–61.

[bb20] Navarrete-Vazquez, G., Cedillo, R., Hernandez-Campos, A., Yepez, L., Hernandez-Luis, F., Valdez, J., Morales, R., Cortes, R., Hernandez, M. & Castillo, R. (2001). *Bioorg Med Chem.* **11**, 187–190. 10.1016/s0960-894x(00)00619-3 11206455

[bb21] Olesen, S. P., Munch, E., Moldt, P. & Drejer, J. (1994). *Eur. J. Pharmacol.* **251**, 53–59. 10.1016/0014-2999(94)90442-1 8137869

[bb22] Ouzidan, Y., Kandri Rodi, Y., Fronczek, F. R., Venkatraman, R., El Ammari, L. & Essassi, E. M. (2011). *Acta Cryst.* E**67**, o362–o363. 10.1107/S1600536810052141 PMC3051530 21523041

[bb23] Rao, A., Chimirri, A., De Clercq, E., Monforte, A. M., Monforte, P., Pannecouque, C. & Zappalà, M. (2002). *Farmaco*, **57**, 819–823. 10.1016/s0014-827x(02)01300-9 12420877

[bb24] Ravina, E., Sanchez-Alonso, R., Fueyo, J., Baltar, M. P., Bos, J., Iglesias, R. & Sanmartin, M. L. (1993). *Arzneim. Forsch.* **43**, 684–694. 8352825

[bb25] Rémond, G., Portevin, B., Bonnet, J., Canet, E., Regoli, D. & De Nanteuil, G. (1997). *Eur. J. Med. Chem.* **32**, 843–868.

[bb26] Saber, A., Sebbar, N. K., Hökelek, T., El hafi, M., Mague, J. T. & Essassi, E. M. (2018*b*). *Acta Cryst.* E**74**, 1842–1846. 10.1107/S2056989018016298 PMC6281085 30574385

[bb27] Saber, A., Sebbar, N. K., Hökelek, T., Hni, B., Mague, J. T. & Essassi, E. M. (2018*a*). *Acta Cryst.* E**74**, 1746–1750. 10.1107/S2056989018015219 PMC6281105 30574367

[bb28] Saber, A., Sebbar, N. K., Sert, Y., Alzaqri, N., Hökelek, T., El Ghayati, L., Talbaoui, A., Mague, J. T., Baba, Y. F., Urrutigoîty, M. & Essassi, M. (2020). *J. Mol. Struct.* **1200**, 127174.

[bb29] Sebbar, N. K., Mekhzoum, M. E. M., Essassi, E. M., Abdelfettah Z., Ouzidan Y., Kandri, Rodi Y., Talbaoui, A. & Bakri, Y. (2016). *J. Mar. Chim. Heterocycl.* **15**, 1–11.

[bb30] Serafin, B., Borkowska, G., Glowczyk, J., Kowalska, I. & Rump, S. (1989). *Pol. J. Pharmcol. Pharm.* **41**, 89–96. 2587441

[bb31] Sheldrick, G. M. (2015*a*). *Acta Cryst.* A**71**, 3–8.

[bb32] Sheldrick, G. M. (2015*b*). *Acta Cryst.* C**71**, 3–8.

[bb33] Spackman, M. A. & Jayatilaka, D. (2009). *CrystEngComm*, **11**, 19–32.

[bb34] Thakurdesai, P. A., Wadodkar, S. G. & Chopade, C. T. (2007). *Pharmacology Online* **1**, 314–329.

[bb35] Turner, M. J., McKinnon, J. J., Wolff, S. K., Grimwood, D. J., Spackman, P. R., Jayatilaka, D. & Spackman, M. A. (2017). *Crystal Explorer 17*. The University of Western Australia.

[bb36] Venkatesan, P., Thamotharan, S., Ilangovan, A., Liang, H. & Sundius, T. (2016). *Spectrochim. Acta Part A*, **153**, 625–636. 10.1016/j.saa.2015.09.002 26452098

